# The Parallel Worm Tracker: A Platform for Measuring Average Speed and Drug-Induced Paralysis in Nematodes

**DOI:** 10.1371/journal.pone.0002208

**Published:** 2008-05-21

**Authors:** Daniel Ramot, Brandon E. Johnson, Tommie L. Berry, Lucinda Carnell, Miriam B. Goodman

**Affiliations:** 1 Program in Neuroscience, Stanford University, Stanford, California, United States of America; 2 Department of Molecular and Cellular Physiology, Stanford University School of Medicine, Stanford, California, United States of America; 3 Department of Biological Sciences, Central Washington University, Ellensberg, Washington, United States of America; Texas A&M University, United States of America

## Abstract

**Background:**

*Caenorhabditis elegans* locomotion is a simple behavior that has been widely used to dissect genetic components of behavior, synaptic transmission, and muscle function. Many of the paradigms that have been created to study *C. elegans* locomotion rely on qualitative experimenter observation. Here we report the implementation of an automated tracking system developed to quantify the locomotion of multiple individual worms in parallel.

**Methodology/Principal Findings:**

Our tracking system generates a consistent measurement of locomotion that allows direct comparison of results across experiments and experimenters and provides a standard method to share data between laboratories. The tracker utilizes a video camera attached to a zoom lens and a software package implemented in MATLAB®. We demonstrate several proof-of-principle applications for the tracker including measuring speed in the absence and presence of food and in the presence of serotonin. We further use the tracker to automatically quantify the time course of paralysis of worms exposed to aldicarb and levamisole and show that tracker performance compares favorably to data generated using a hand-scored metric.

**Conclusions/Signficance:**

Although this is not the first automated tracking system developed to measure *C. elegans* locomotion, our tracking software package is freely available and provides a simple interface that includes tools for rapid data collection and analysis. By contrast with other tools, it is not dependent on a specific set of hardware. We propose that the tracker may be used for a broad range of additional worm locomotion applications including genetic and chemical screening.

## Introduction

The soil nematode *Caenorhabditis elegans* is widely used to study the genetic basis of behavior and other aspects of neurobiology. Advantages of this animal include a fully-sequenced genome, a short generation time, inexpensive and simple methods for laboratory cultivation, advanced techniques in both classical and molecular genetics, and a compact nervous system composed of only 302 neurons. *C. elegans* is the only animal for which the morphology and synaptic connections of the entire nervous system have been reconstructed from electron micrographs [1]. It is also an emerging model for drug discovery (reviewed in [2], [3]) and soil toxicity testing (*e.g.* [4], [5]).

Locomotion has been used extensively to study aspects of neurobiology in *C. elegans* and other nematodes. All nematodes move in a sinuous fashion, by propagating waves of alternating dorsal and ventral contraction. Traditionally, the community of *C. elegans* researchers has relied on observation to quantify differences in locomotion between wild-type and mutant animals and under different conditions (*e.g.* crawling on or off food). Common observational assays take the form of counting the number of body bends per unit time, which serves as a proxy for average speed, and counting the number of animals paralyzed by drugs such as aldicarb and levamisole. Aldicarb is an acetylcholine (ACh) esterase inhibitor that causes ACh to accumulate in the neuromuscular junction. Levamisole is an ACh receptor agonist. Both compounds cause over-stimulation of the body wall muscle and induce rigid paralysis. Screens for mutants that are resistant or hypersensitive to these drugs have revealed genes that regulate synaptic transmission in *C. elegans* [6]–[13]. Many of these genes are conserved in mammals and encode proteins that play essential roles in synaptic transmission [14].

We reasoned that a variety of studies would be enhanced by automated methods for measuring average speed and the fraction of animals paralyzed. Compared to observational methods, automated behavioral analysis is less susceptible to experimenter bias and is likely to yield results that can be compared across experimenters. As noted by Mahoney, *et al.* [15], several factors affect the reproducibility of aldicarb-induced paralysis assays, including the criteria used to score paralysis. For example, researchers are urged to assay all genotypes in parallel, which severely limits the number of genotypes that can be investigated. Our goal was to develop a simple platform compatible with a variety of digital video cameras and stereomicroscopes that could track tens of animals in parallel, extract key parameters of locomotion and replace observational measures of average speed and drug-induced paralysis. To facilitate wide use of the tracker in the research community, we chose to implement our tracker in MATLAB® (The MathWorks, Natick, MA), a platform that is used in both science and engineering, to distribute the code freely (http://wormsense.stanford.edu), and to foster further community-based development by creating an open-source project for it on SourceForge (http://sourceforge.net/projects/wormtracker).

Existing trackers can be classified according to the information extracted from video frames about worms: the centroid position [16]–[19] or a curve corresponding to the central “skeleton” of the worm's image [20]–[25]. All of these trackers rely on high contrast images, which can be generated by transmitted or oblique (dark-field) illumination and a simple microscope. Centroid-based trackers define worm position as the geometric center of a rectangular box that encloses the worm's image in each video frame [16]–[18]. They can follow multiple animals at low magnification or, with the aid of a motorized x-y stage and feedback control, they can follow single animals over minutes or hours [17], [19]. The throughput of such trackers can be increased by operating several setups in parallel, as reported by Shtonda and Avery [26]. Centroid-based trackers provide limited information about the details of worm posture and cannot easily distinguish between forward and reverse movement. Skeleton-based trackers, by contrast, generally operate at high (40–60×) magnification and derive a skeleton of each worm from segmented binary images [20]–[22], [25]. These skeletons provide extensive information about posture, and skeleton-based trackers have been used to classify mutants that disrupt locomotion [23], [24]. Most [20]–[22], but not all such trackers [25] rely on a particular motorized x-y stage and are limited to analyzing single worms. (Algorithms for skeleton-based multi-worm trackers are emerging in the literature, however [27]–[30].)

Here, we describe a parallel worm tracker, implemented in MATLAB®, which records the centroid position of tens of worms in sequential video frames, terminating tracks when animals collide. Tracks are used to compute worm speed and angular velocity. One application of these metrics, which we have described elsewhere [31], is automatic detection of turning events known as pirouettes. Here, we adapt the parallel worm tracker to determine worm speed and to derive a measure of the fraction of worms that are paralyzed by drug application. If the animal density is not too high (for example, <15 young adult worms per square cm) and individuals are assumed to be indistinguishable, we show that this simple approach is sufficient to capture essential aspects of locomotion and its modulation by drugs, gene mutations, or both. We support this claim by comparing aldicarb-induced paralysis measured by the tracker and by manual scoring.

## Methods

### Nematodes

Synchronized populations of wild-type and mutant nematodes were prepared by standard methods and cultivated at 20°C on NGM agar containing *E. Coli* (OP50) [32]. Worms were assayed as young adults. The following worm strains were used: wild type (N2, Bristol) and NM1968 *slo-1(js379)* V. We transferred worms to assay plates as follows. First, 30–50 worms were washed from culture plates in normal physiological saline [33] and centrifuged briefly (30 s) to concentrate worms. Next, 10–20 µl of solution containing concentrated animals were pipetted onto a stack of four 0.5 cm filter paper disks (cut from Whatman, No. 1 with a standard one-hole punch). Finally, the top disc was inverted on the assay plate, which results in the transfer of ∼25 worms. This technique is an effective method for rapidly transferring a large number of animals without scratching the agar surface (important for obtaining high-contrast videos). Picking animals to the assay plate also works well, albeit is typically slower.

### Assay plates

To prepare assay plates, we poured 3 ml NGM into 35 mm Petri plates one day prior to tracking worms. Aldicarb, levamisole, and serotonin were added to the molten agar to yield final concentrations of 1 mM, 400 µM and 7.5 mM, respectively. We discarded plates that contained particles that could interfere with tracking. To make assay plates with OP50, we concentrated a liquid culture of OP50 six times, spread 20 µl over their surface, and dried the plates for 15 minutes.

### Chemical corral

Worms were confined to the field of view using a copper chloride corral. The corral consisted of a rectangular filter paper frame (Whatman No. 1) with inside dimensions of 2.0×1.7 cm saturated with 100 mM CuCl_2_. This chemical corral is effective only for worms that avoid Cu^2+^, a behavior which requires the ASH neurons [34].

### The parallel worm tracker

MATLAB® source code for the parallel worm tracker and a User Manual are available for download at http://wormsense.stanford.edu/tracker/, as supplemental material (Dataset S1, Text S1, Text S2), and as part of an open-source development project at http://sourceforge.net/projects/wormtracker. A video of the tracker in action (Movie S1) illustrates the large number of worms that can be tracked in parallel. The worm tracker automatically identifies worms and tracks their position as described previously [31]; position is defined as the worm's centroid (center of mass). The tracker also maintains information about the size and shape (eccentricity) of the worms in each movie frame. Tracking is performed off-line, after video capture has been completed. The worm tracking software is compatible with a variety of video cameras and microscopes. The key requirements are MATLAB®, the MATLAB® Image Processing Toolbox™ and a video camera and microscope that permit capture of high-contrast videos. (MATLAB® and the Image Processing Toolbox™ are available for most commonly used operating systems, including Windows, Mac OS X, and Linux; for a complete list please see the Mathworks website.) The tracker is designed to analyze video stored in AVI format, and has been tested with uncompressed, grayscale (8-bit) movies at a resolution of 640×480 pixels. It may be necessary to convert movies stored using alternative formats or captured at a different resolution into the format specified above; this can be easily achieved using commercially available video-editing software.

**Figure 1:**
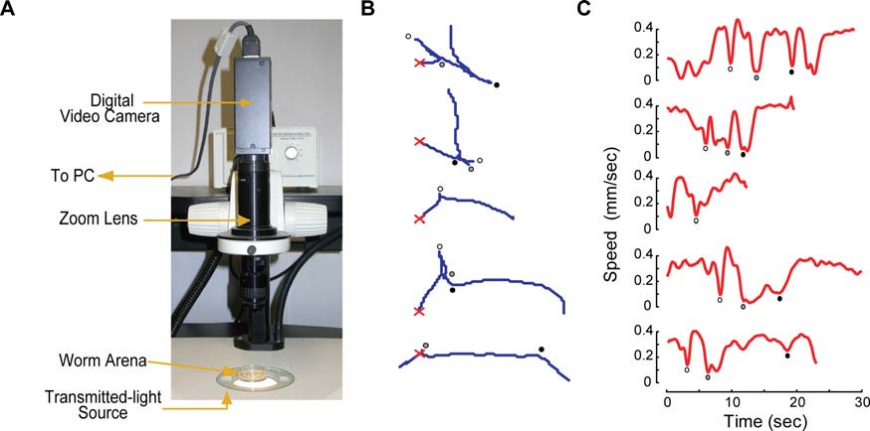
Worm tracker apparatus and representative worm tracks. (A) Photograph of an apparatus for imaging and tracking worms. (B) Representative tracks generated by the worm tracker. The red×marks the worm's starting position. Track lengths were (top to bottom): 7.4, 5.4, 3.6, 8.0 and 6.6 mm. (C) Worm speed, as measured by the tracker for the tracks in B. Filled circles denote turning events; corresponding turns in B and C are marked using circles of the same color.

In addition to the parallel worm tracker, the package includes software for directly capturing tracker-compatible video using MATLAB®. This software requires the MATLAB® Image Acquisition Toolbox™ (as of this writing, this Toolbox is only available for 32-bit versions of Windows) and use of a camera supported by this toolbox; a complete list of supported capture devices is available on the Mathworks website and includes all digital cameras compatible with the IIDC 1394-based Digital Camera Specification (DCAM). Though the code published here is written for a DCAM-compatible camera, it can be adjusted to any video format supported by MATLAB® (see User Manual, Text S1). The system we use is illustrated in Fig. 1 and consists of a transmitted light base (TLB 3.1, Diagnostic Instruments, Inc., Sterling Heights, MI), a zoom lens with C-mount adaptor and 0.5× lens (Navitar, Rochester, NY), a DCAM-compatible video camera (XCD-900, Sony), and a desktop PC (Optiplex 745, Dell) running Windows XP.

A companion software package, WormAnalyzer, provides tools for analysis and display of tracks generated by the worm tracker. WormAnalyzer computes the speed and angular speed of worms and automatically discriminates between runs and turns (pirouettes) using an angular speed threshold [17], [31]. These data are then used to measure average speed and to estimate the fraction of worms paralyzed in each video. Note that speed and angular speed computations rely on several parameters that are setup-specific, such as camera frame rate and image magnification; these parameters must be set to the appropriate values through the WormAnalyzer user interface, as described below (also see Text S1).

### Criteria for paralysis

Worm tracks were associated with paralyzed worms if 80% of the instantaneous speed measurements collected during the track (one speed measurement per video frame) were less than 0.015 mm/s. To account for the variability in track durations, the ‘Fraction Paralyzed’ is computed by dividing the total duration of all paralyzed tracks by the total duration of all tracks in the experiment. Paralysis criteria were chosen to match our hand-scored data, but can be easily adjusted (see User Manual, Text S1). To facilitate comparison among research reports, we suggest that groups using the tracker to measure paralysis report the exact values of these parameters.

### Tuning the tracker

There are a small number of parameters that must be tuned by hand for each setup; correct tuning is critical because of differences in the camera, lighting, zoom, etc. and the worms under study. Once configured, however, there is no need to re-tune these parameters as long as the setup is not altered. All parameters can be directly accessed and set through the worm tracker user interface. Directions and tips for tuning parameters, and a description of tracker features specifically designed to facilitate tuning, are provided in the User Manual (Text S1). The parameters are:

1. Threshold intensity for converting grayscale movie frames into binary images. Conversion of movie frames into binary images is the first step of the image segmentation process applied to each frame. Values must be between 0 (black) and 1 (white). A starting value is calculated automatically for each frame, based on image statistics of that frame. This allows for moderate lighting changes throughout the movie. However, in our experience it is often necessary to apply a small, user-defined offset to this value to ensure reliable thresholding.
2. The minimum and maximum size (in total pixels) of an object that will be identified as a single worm. These values should be selected so that objects smaller than a single worm and larger than clusters of two or more worms are ignored by the tracker. These parameters need to be tuned according the population of animals under study, camera resolution and zoom. For example, values appropriate for populations of L1 larvae will be smaller than those for adult animals. Though it is possible to assay mixed stage animals, doing so is likely to degrade the accuracy of the tracker. The tracker uses a size criterion to identify worms and because it is likely to be difficult, if not impossible, to establish size thresholds for mixed populations that reliably exclude both clumps of multiple worms and spurious objects, such as dust specks, that are smaller than single worms.

The parameters listed below can also be tuned manually, although the default values should work well in most cases. We recommend altering these values only if the tracker is not functioning properly.

1. Maximum distance traveled by worms between successive frames. A newly identified worm-object is associated with an existing track only if the distance between the new centroid and that of the existing track is smaller than this threshold. A value of five pixels typically works well, but the best value may depend on microscope magnification, camera pixel size and frame rate.
2. Minimum valid track duration. Tracks shorter than this threshold are discarded. This is useful to avoid tracking spurious objects. Values between 50 and 100 frames (between 6.7 and 13.3 s at our camera's frame rate) usually work well. The default value is 100 frames.
3. Maximum size change between successive frames. Objects whose size changes by more than this threshold between two consecutive frames cannot be considered part of the same track. This is a useful mechanism for identifying collision events between two worms. The default value is 100 pixels.

## Results

**Figure 2:**
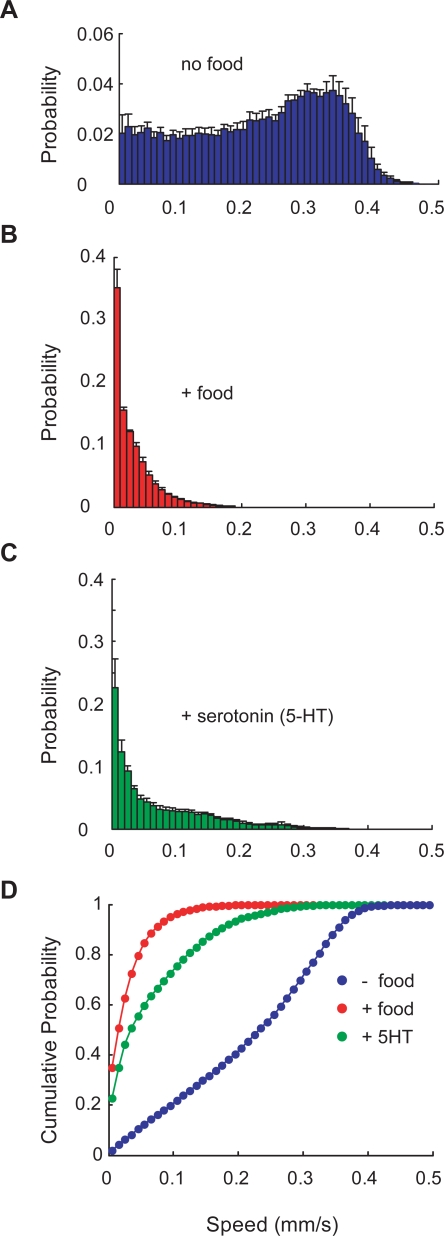
The effect of bacterial food and serotonin (5-HT) on worm locomotion. Distributions of worm speeds measured on (A) NGM only (no food); (B) NGM+food (*E. coli* OP50); and (C) NGM+5-HT. In each experiment, approximately 20 worms were tracked for 30 s. Histograms are the average of five experiments. Error bars are s.e.m. In all cases, worms were tracked 30 minutes after they were transferred from their cultivation plates to the experimental plate. (D) Cumulative probability distributions derived from the plots in A–C.

We used the parallel worm tracker to extract two simple measures of locomotion: average speed and the fraction of paralyzed worms. To test the utility of the system, we determined the effect of bacterial food (*E. coli* OP50) and serotonin (5-hydroxytryptophan) on the average speed of wild-type (N2) adult hermaphrodites. Consistent with prior reports [18], [26], [35], we found that both treatments reduced average speed (Fig. 2). Although the distribution of speeds overlap in the three conditions (Fig. 2), the parallel worm tracker can easily collect data sets large enough to reliably resolve differences in average speed (Fig. 2D). Average speeds in the presence and absence of food were comparable to those reported by others (Table 1). We note, however, that values reported in the literature are variable. The origin of this variability is unknown, but may reflect differences among strains, variations in experimental parameters, differences in the tracking algorithms deployed, or a combination of these factors.

**Table 1:** Average speed of wild-type (N2, Bristol) worms.

Substrate	Average Speed (µm/s)	Reference
NGM+2 mM NH_4_Cl	152	[17]
NGM	232	[18]
NGM	250	[39]
NGM	165	[40]
NGM	16	[41]
NGM	180	[42]
NGM	120	[43]
NGM	219±29*	*This study*
NGM+food	109	[18]
NGM+food	82	[43]
NGM+food	34	[44]
NGM+food	79	[45]
NGM+food	120	[46]
NGM+food	34	[47]
NGM+food	15.9	[48]
NGM+food	92.4	[49]
NGM+food	31±4*	*This study*

*mean±s.d.

**Figure 3:**
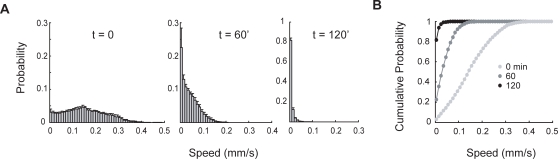
Response of wild-type (N2) worms to aldicarb. (A) Speed distributions of worms crawling on NGM containing 1 mM aldicarb at three time points. In each experiment, approximately 20 worms were tracked for 30 s. Histograms are the average of five experiments. Error bars are s.e.m. (B) Cumulative probability distributions derived from the data shown in A and similar experiments at three additional time points.

Mutations and certain drugs that disrupt synaptic transmission lead to paralysis. Screens for mutations that suppress drug-induced paralysis have revealed genes required for the synthesis of neurotransmitters, packaging neurotransmitters into synaptic vesicles, vesicle release, and receptor function [14]. We reasoned that the tracker could be an effective tool for measuring drug-induced paralysis and tested this idea by using the tracker to measure the response to aldicarb, an acetylcholinesterase (AChE) inhibitor, that causes ACh to accumulate in the neuromuscular junction and induces hyper-contraction and paralysis. As expected, the distribution of worm speeds shifts toward small values during exposure to aldicarb (Fig. 3).

**Figure 4:**
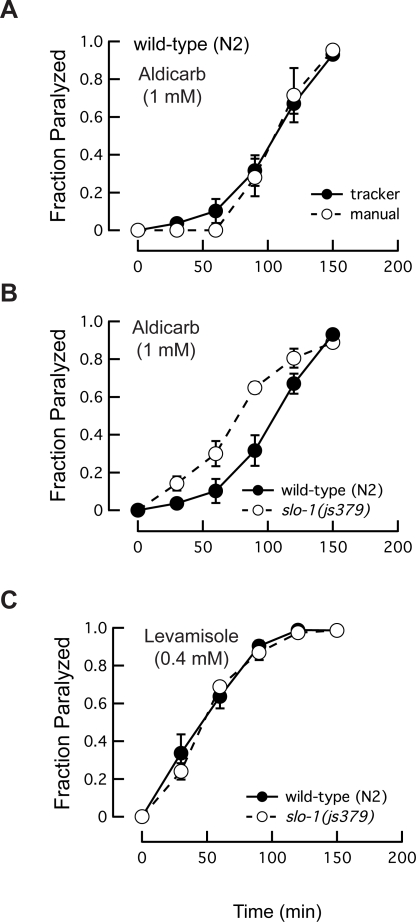
Responses of wild-type (N2) and *slo-1* mutant worms to aldicarb and levamisole. (A) Comparison of the worm tracker and manual scoring of paralysis for wild-type and *slo-1* worms crawling on NGM containing 1 mM aldicarb. (B) Comparison of the time course of aldicarb-induced paralysis of wild-type and *slo-1* worms. (C) Comparison of the time course of levamisole-induced paralysis of wild-type and *slo-1* worms. Data in B and C were obtained using the worm tracker. In all three panels, points are the average fraction of paralyzed tracks observed at each time point (*n* = 5 assays). Bars are s.e.m.

From these distributions, we estimated the fraction of worms paralyzed (see Methods) and compared the results to assays in which paralysis was scored manually (Fig. 4A). The average time to 50% paralysis (*T*_50_) was essentially identical in both assays, indicating that the parallel worm tracker is a reliable and efficient technique for assaying the effects of drugs on locomotion. The *T*_50_ values we report are higher than those reported in the literature (Table 2), however. This suggests that we applied especially stringent criteria for paralysis. It is noteworthy that *T*_50_ values obtained by observation are highly variable both among and within published reports.

**Table 2:** Aldicarb-induced paralysis in wild type (N2, Bristol) worms.

*T*_50_ (min)	Method	Reference
105	Manual	*This study*
106	Tracker	*This study*
77	Manual	[15]
70	Manual	[36]
62	Manual	[50]
56	Manual	[51]
71	Manual	[52]
69	Manual	[53]
48, 58, 78	Manual	[54]
68	Manual	[55]
109	Manual	[56]
60, 52	Manual	[57]
50	Manual	[58]
73	Manual	[59]

As a further demonstration of the utility of the tracker, we compared paralysis induced by exposure to aldicarb and levamisole in wild-type and *slo-1* null mutant worms. The *slo-1* gene encodes a large-conductance calcium- and voltage-gated K^+^ channel expressed in motor neurons and body wall muscle [36]. Mutations that alter aldicarb sensitivity may have a pre- or post-synaptic effect on synaptic transmission, whereas mutations that alter levamisole sensitivity most likely have a post-synaptic effect. As reported previously [36], *slo-1* mutants were hypersensitive to aldicarb-induced paralysis (Fig. 4B). Consistent with the idea that regulation of transmitter release is the dominant function of SLO-1 K^+^ channels, loss of *slo-1* had no detectable effect on levamisole-induced paralysis (Fig. 4C).

## Discussion

The parallel worm tracker is a flexible, low-cost solution for studying *C. elegans* locomotion and drug-induced paralysis. It uses a centroid-based algorithm to extract two fundamental parameters of locomotion: instantaneous speed and angular speed. Previously, we used angular speed to automatically detect turning events known as pirouettes [31]. Here, we show that the parallel worm tracker can measure average speed as well as drug-induced paralysis. As evidenced by the recent proliferation of tracking systems geared toward investigations of *C. elegans* locomotion [20]–[25], [27]–[30], [37], [38], automated methods for scoring behavioral phenotypes offer several advantages over manual methods. Using the tracker to measure average speed, for example, is faster and less susceptible to unconscious observer bias than classical measures of locomotion rate such as observing and recording the number of body bends executed per unit time. Tracker-based scoring of drug-induced paralysis is more reproducible and less subjective than manual scoring. This should facilitate comparisons among assays conducted by different observers within one laboratory or among several laboratories. It also sets the stage for developing databases that link quantitative measures of behavior (phenotype) to genotypes in *C. elegans*. Finally, observational methods are labor-intensive and limit the number of animals, genotypes, and conditions that can be investigated. As currently configured, the parallel worm tracker can assay ∼5 replicates of groups of 10–30 animals in a few hours. Compared to other systems, the MATLAB®-based parallel worm tracker is compatible with a wide variety of video cameras and microscopes, reducing the need to purchase new hardware. Additionally, since MATLAB® is widely used in academic research, users can easily customize or modify the tracker for other applications and many will have access to university-based site licenses for MATLAB®. Finally, the parallel worm tracker could be integrated into a high-throughput assay system similar to those deployed in cell-based high-content screening (HCS) in which MATLAB® is used to control a plate-handling robot and motorized x-y stage as well as data acquisition and analysis.

## Supporting Information

Dataset S1 MATLAB-based code for the Parallel Worm Tracker. This *.zip file contains all of the *.m files needed to run the Parallel Worm Tracker. It also contains the user manual and an Excel file used to define run-time preferences.
(0.36 MB ZIP)

Text S1 Parallel Worm Tracker/Track Analyzer - Version 2.0 (February 2008). A user manual for the MATLAB-based parallel worm tracker.
(0.29 MB PDF)

Text S2 Block Diagram of a portion of the MATLAB-based code. This block diagram is for the Track Analysis package and is intended as a guide for users who wish to modify the code.
(0.04 MB PDF)

Movie S1 Worm tracking movie. Illustrates tracker performance and typical contrast needed for effective tracking. Centroids are marked with a blue cross; tracks are shown in red and end when animals collide or leave the field of view
(0.00 MB MOV)
