# Supplementary material for: The Parallel Worm Tracker: A Platform for Measuring Average Speed and Drug-Induced Paralysis in Nematodes
Source: PLoS One. 2008 May 21;3(5):e2208. doi: 10.1371/journal.pone.0002208 (PMC2373883; doi:10.1371/journal.pone.0002208)
Supplement: Dataset S1 — MATLAB-based code for the Parallel Worm Tracker. This *.zip file contains all of the *.m files needed to run the Parallel Worm Tracker. It also contains the user manual and an Excel file used to define run-time preferences. (0.36 MB ZIP) [file pone.0002208.s001.zip › Parallel Worm Tracker User Manual.pdf]

Worm Tracker & Track Analyzer – Version 2.0 (February 2008)
User Manual

I. Copyright Notice & Disclaimer:

This source code (software) is distributed through the Department of Molecular and Cellular Physiology at Stanford University and made freely available for research purposes only. The copyright is retained by the authors.

Copyright © 2008, Daniel Ramot, Miriam B. Goodman. All rights reserved.

The use of this software is subject to the following conditions:

(1) The package and portions thereof may not be sold. Users may modify the software for their own use, but may not redistribute any version of the software other than the original version without the express permission of the author and Stanford University.
(2) This software package is provided on an "as is" basis. The author in no way warrants either this software or results it may produce. Use of this software is at your own risk.
(3) The author is under no obligation to provide any services by way of maintenance, updates or corrections for this software.
(4) Reports or publications resulting from use of this software package must contain an acknowledgment in the form commonly used in academic research.

II. Brief Description of the Software:

The software is composed of three modules: a worm tracker; an analysis environment for viewing and analyzing tracks generated by the tracker; and a video capture utility.

A) *The Worm Tracker*: automatically identifies and tracks multiple worms in a movie. Each movie frame is segmented into objects – regions of contiguous pixels that are all darker (or brighter – this can be selected by the user) than a threshold. Certain properties, including centroid coordinates, size and eccentricity are calculated for each object. Objects are identified as single worms by size criteria. Tracks are generated by linking objects whose centroids are closest to one another and not further than a threshold value in successive frames. In this manner, worms are tracked from frame to frame, and their properties are logged to a Tracks File, which is the output of the tracker. When worms "collide" their combined size exceeds a size threshold, indicating to the tracker that the object contains more than a single worm, and their tracks are terminated. When worms part ways after a collision event, they are once again identified by the tracker as valid single worms, and new tracks are created for them. Movie files are designated for tracking using a simple user interface, which also allows the user to adjust tracker parameters.

B) *The Worm Analyzer*: provides a user-friendly environment for viewing and analyzing tracks generated by the worm tracker. The Worm Analyzer contains a "player" function, which allows tracks to be displayed superimposed on the original movie and played back. Each worm track can be analyzed to calculate worm speed, angular speed and direction of locomotion for the duration of the track. This analysis automatically identifies turning events (reversals and omega bends), known as pirouettes. A description of the algorithm for identifying pirouettes is provided

in the Analysis Preferences section. All tracks associated with a movie can be analyzed in batch mode, and the analysis results saved to a file. This file can then be read by another analysis function, also operated via the Worm Analyzer window, and used to calculate pirouette initiation probabilities throughout the experiment. This can be useful for analyzing the effect of a stimulus, for example an increase in the concentration of a chemical attractant, on worm turning behavior. In addition, probability distributions of worm speeds and the fraction of paralyzed worms can be computed and plotted for individual movies or for data pooled from multiple movies.

C) *Video Capture*: provides a user-friendly interface for capturing movies using MATLAB and for controlling basic video camera parameters (gain, shutter speed). Movies are directly captured and encoded in a format supported by the Worm Tracker. The Video Capture utility can be used to capture a sequence of movies at regular intervals, providing a means for collecting multiple brief video samples over an extended period of time. In this mode, newly captured videos can be automatically tracked by the Worm Tracker between capture periods, expediting the data analysis process.

Note: A basic level of familiarity with MATLAB is necessary for using the software.

III. Installation Instructions (PLEASE FOLLOW CAREFULLY):

1. Download and unzip the MATLAB functions into the directory 'C:\Matlab Functions' (if this directory does not exist on your computer, it must be created; the tracker will not function properly if its functions are not located within this directory).
2. When the functions are unzipped, three new directories should be created: Worm Tracker, Video Capture and Track Analysis (which includes a subdirectory, Playback Track). Add the three new directories and the subdirectory to MATLAB's path (in MATLAB's command window, select: File → Set Path… and add the relevant folders). You should also find an Excel file, 'Worm Tracker Preferences.xls', in the 'Matlab Functions' folder. This file should not be removed from the 'Matlab Functions' directory, as it contains all of the software's default preferences and is loaded from this folder each time the software is initialized.

IV. Compatibility:

This software package was written in MATLAB® (© The MathWorks, Inc., http://www.mathworks.com). Worm Tracker and Worm Analyzer require the MATLAB environment with the Image Processing Toolbox™ installed to run; Video Capture also requires the Image Acquisition Toolbox™.

The software was written using MATLAB Release R2007a on a PC running WindowsXP, and has not been tested on other operating systems; some modifications may be necessary for the code to function properly on a different platform. (Note: As of this writing (February 2008), the Video Capture software is not compatible with Windows Vista.)

The Worm Tracker and Worm Analyzer utilities are designed to work with *uncompressed, grayscale (8-bit) movies in AVI format with a resolution of 640x480*. Movie file names must end with the .avi extension. You may need to modify the tracking and analysis software for other movie formats or resolutions. Movies can be converted into the required format using standard, commercially available movie editing software, such as VideoMach (© www.gromada.com).

Video Capture is written to work with a Sony XCD-SX900 digital camera, storing uncompressed, 640x480 grayscale (8-bit) movies in AVI format. Since the XCD-SX900 camera captures images at a resolution of 1280x960 pixels, Video Capture uses MATLAB's imresize function to reduce image resolution to 640x480 during capture. Instructions for configuring Video Capture for different cameras are provided in the Video Capture Preferences section.

V. The Worm Tracker:

To open the Worm Tracker interface (shown in Fig. 1) type WormTracker in the MATLAB command window.

The tracker can be set to track multiple movies. Enter up to five full movie names (including directories) into the boxes entitled Name. For example, in Fig. 1, the entry for Movie 1 will initiate tracking of the movie 'N2 Experiment.avi' located in the folder 'C:\Movies' (note this will also work if the .avi extension is omitted). To track a sequence of movies whose names differ only by a serial number attached to the end of the movie name, enter the first and last serial numbers in the boxes entitled Start and End. For example, the entry for Movie 2 initiates tracking of the movies 'N2 Experiment_1.avi', 'N2 Experiment_2.avi' and 'N2 Experiment_3.avi' located in the folder 'C:\Movies'.

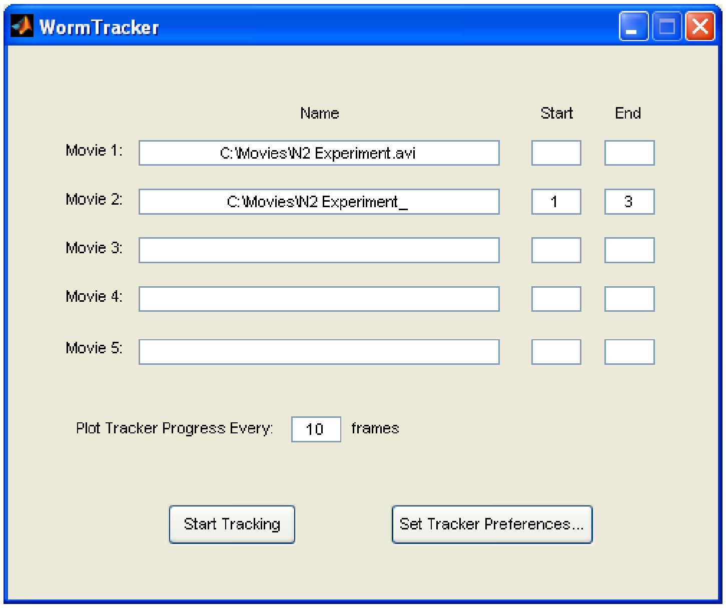

Figure 1: The Worm Tracker interface

To begin tracking, press the 'Start Tracking' button. The tracker will track the designated movies in order, and display its progress periodically (Fig. 2); the period of display is determined via the 'Display Tracker Progress Every' field (Fig. 1); the default is 10 frames.

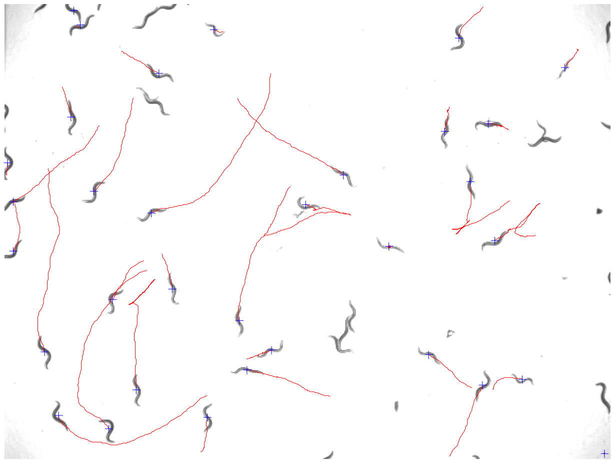

Figure 2: The Worm Tracker in action. Blue crosses are centroids of identified single worm objects; red lines indicate "live" tracks

Tracking results are saved in the form of a Tracks File. Tracks Files are assigned the same name as the movie that was used to generate them, but end in .mat in place of .avi.

Tracking tip: Good contrast is key! Movies with poor contrast between worms and the background inevitably result in poor tracker performance.

Tuning the tracker (adjusting tracker preferences): For the tracker to function properly, a small number of setup-specific parameters must be tuned by hand; correct tuning is critical because of differences in the camera, lighting, zoom, etc. and the worms under study. Once configured, however, there is no need to re-tune these parameters as long as the setup is not altered. All parameters can be directly accessed by pressing the 'Set Tracker Preferences' button in the Worm Tracker user interface (Fig. 1). Pressing this button opens the interface shown in Fig. 3. There are two sets of parameters that must be tuned:

Image Segmentation Parameters: Conversion of grayscale movie frames into binary images is the first step of the image segmentation process applied to each frame. The threshold intensity for conversion to binary images is critical for properly distinguishing objects from the background, and often requires manual tuning. Threshold values are between 0 (black) and 1 (white). To automatically calculate a starting value for each frame based on image statistics of that frame[1], select 'Use Auto Thresholding' (the default option). This allows for moderate lighting changes throughout the movie. However, in our experience it is often necessary to apply a small offset to this value to ensure reliable thresholding; adjust this offset via the 'Auto Thresholding Correction Factor' box. To set a constant threshold for all movie frames, uncheck the 'Use Auto Thresholding' box, and enter a value in the 'Manual Threshold' box. Also use the Worm Tracker Preferences interface to select the tracking mode (dark worms on a white background or white worms on a dark background).

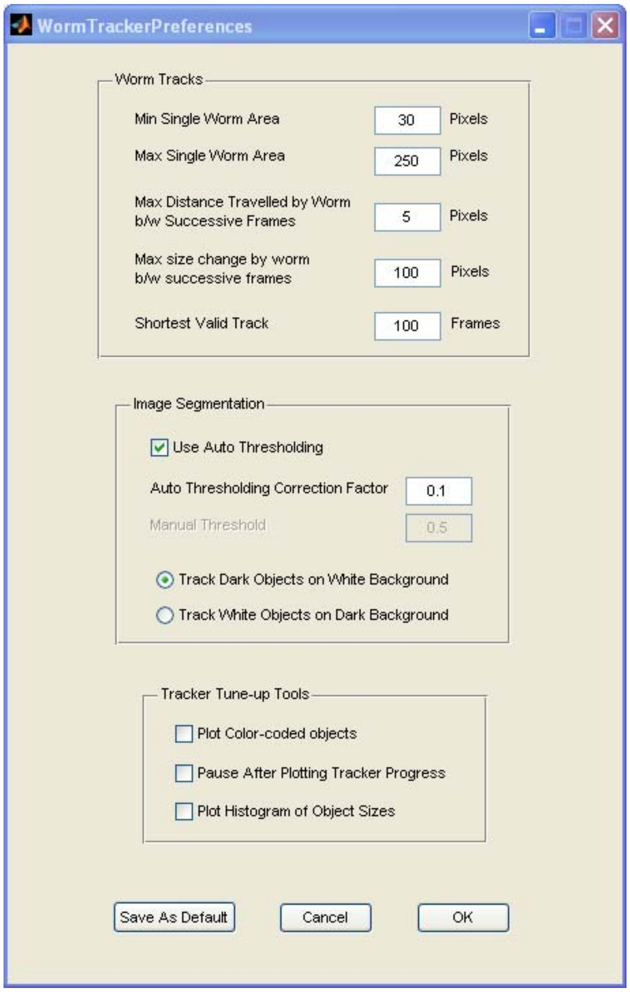

Figure 3: Interface for adjusting Worm Tracker preferences

Worm Tracks Parameters: To ensure that the Worm Tracker identifies single worms as valid objects and ignores other objects, the parameters 'Min Single Worm Area' and 'Max Single Worm Area' may require tuning. These parameters determine the minimum and maximum size (in total pixels) of objects that will be classified as single worms. These values should be selected so that specks smaller than a worm and clusters of two or more worms are

[1] A good (and fairly brief) introduction to thresholding, and an explanation of the method used by Matlab's 'graythresh' function to calculate this starting value (Otsu's method) can be found at: http://homepages.inf.ed.ac.uk/rbf/CVonline/LOCAL_COPIES/MORSE/threshold.pdf

ignored by the tracker. These parameters will need to be tuned according the population of animals under study, camera resolution and zoom. For example, values for populations of L1 larvae will be smaller than those for populations of adult animals.

Three additional Worm Tracks parameters can be adjusted manually, although the default values should work well in most cases. We recommend altering these values only if the tracker is not functioning properly.

1. Maximum Distance Traveled by Worm between Successive Frames: A newly identified worm-object is associated with an existing track only if the distance between the new centroid and that of the existing track is smaller than this threshold. A value of 5 pixels typically works well, but the best value may depend on microscope magnification and camera pixel size and frame rate.
2. Maximum Size Change by Worm between Successive Frames: Objects whose size changes by more than this threshold between two consecutive frames cannot be considered part of the same track. This is a useful mechanism for identifying collision events between two worms. The default is 100 pixels.
3. Shortest Valid Track: Tracks whose duration is shorter than this threshold are discarded. This is useful to avoid tracking spurious objects that are sometimes identified by the segmentation algorithm, but typically do not last for more than a few frames. Values between 50 and 100 frames (between 6.7 and 13.3 s at our camera's frame rate) usually work well. The default value is 100 frames.

Tracker Tune-up Tools: The tracker features three options that enhance the tracker's progress display, and can be activated to facilitate tuning:

1. Plot Color-Coded Objects: Select this box to display, in addition to the standard tracker progress frame (Fig. 2), a color-coded map of *all* objects detected by the tracker (before the 'Min Single Worm Area' and 'Max Single Worm Area' size thresholds are applied). White circles mark the centroids of objects identified as single worms using the size thresholds. This tool provides a means to assess the performance of the segmentation process and the size criterion used to identify worms. For example, if a single worm is being segmented into two distinct objects because of an incorrect intensity threshold, the worm will appear to have two different colors in this plot. In that event, it may be necessary to adjust the 'Auto Thresholding Correction Factor' and re-run the tracker.
2. Pause After Plotting Tracker Progress: Activating this option instructs the tracker to wait for a keyboard stroke after each progress update plot before resuming tracking. This allows additional time to examine the progress plot during the tracker tuning process.
3. Plot Histogram of Object Sizes: Provides an additional plot during progress updates, which can be used to tune the 'Min Single Worm Area' and 'Max Single Worm Area' thresholds.

Updating Default Preferences: Once tracker parameters have been tuned, save their values using the 'Save As Default' button. Pressing this button saves all current preferences to the 'Tracker Prefs' worksheet in the Excel file 'Worm Tracker Preferences.xls', located in the 'Matlab Functions' folder. (*Note*: the file must be closed when the 'Save As Default' button is pressed, or MATLAB will not be able to access it.) Default preferences can also be directly modified in 'Worm Tracker Preferences.xls' using Excel. We do not recommend this method however, as changes to the spreadsheet may result in tracker failure.

VI. The Analysis Environment (Worm Analyzer):

To start the analysis environment, type WormAnalyzer in the MATLAB command window. All track analysis commands appear under the 'Tracks' menu, located to the right of the 'Help' menu.

Opening Tracks Files: Tracks files (the output of Worm Tracker) can be opened using the 'Open Tracks File' command. After clicking on the 'Open Tracks File' option in the 'Tracks' menu, you will be prompted to select a Tracks file. You will then be asked to indicate how many movies are associated with this Tracks file (1 or 2 - see note below), and to select these movies. The selected movie(s) will be displayed, with individual tracks superimposed.

Note: the option for two movies per Tracks file is the result of a MATLAB file size limit of 2GB. To allow capture and analysis of movie files up to 4GB, Video Capture splits such movies into two files, each up to 2GB. Use Worm Tracker to track both movies and generate a Tracks file for each of them, then combine the two Tracks files into a single Tracks file using the JoinTracks function (requires setting Tracks and movie filenames directly in the JoinTracks code). When viewing the combined Tracks file in Worm Analyzer, you will need to select both movies.

Viewing Tracks: Tracks are displayed one at a time. Scroll through all of the tracks in the open Tracks file using the scroll bar at the top right corner of the analysis window.

Deleting Invalid Tracks: Tracks deemed to be invalid (dirt specks incorrectly identified as worms, etc.) can be deleted from the Tracks file using the 'Delete Track' button at the top right corner of the analysis window. When you are done deleting invalid tracks, use the 'Save Tracks File' option under the 'Tracks' menu to save an updated Tracks file.

Playing Back Tracks: Tracks can be played back using the buttons at the top left corner of the analysis window. As a default, the fast forward (and rewind) play rate is set at 6x the normal frame rate (and can be adjusted via the Preferences menu). Frame number and time are continuously updated during playback and displayed above the movie.

Analyzing Single Tracks: Scroll through the tracks in the Tracks file to select the track you would like to analyze, and then select the 'Analyze Current Track' option in the 'Tracks' menu. Analysis results are displayed in a separate figure. The worm's instantaneous direction, speed and angular speed, if selected in the Preferences window, are plotted against time for the duration of the track. Pirouettes are automatically identified, and their duration marked in green on both the angular speed trace and the track displayed in the main analysis window. Analyzing a track also updates its display, replacing the raw track with smoothed track coordinates.

Analyzing All Tracks in an Experiment: Select the 'Analyze All Tracks' option in the 'Tracks' menu to analyze all tracks in the open Tracks file. Analysis results are not displayed, but saved to a file – the user is prompted to input the file name.

Computing Pirouette Probabilities: To compute pirouette initiation probabilities, select the 'Display Pirouettes' option under the 'Tracks' menu. To apply this analysis to a particular movie you must first analyze all tracks associated with that movie (see above). When prompted to select a Tracks file for analysis, select the file you saved after analyzing all tracks (not the Tracks file originally generated by WormTracker). Pirouette initiation probabilities are computed by normalizing pirouette initiation events as described by Pierce-Shimomura et al. (1). Briefly, for each movie frame the function calculates: *a*. The number of worms being tracked; *b.* The number of tracked worms that could initiate a pirouette (i.e. worms that are not already turning or turned

less than ‘Min Run Duration’ seconds ago); *c.* The number of pirouettes initiated; and *d.* The instantaneous pirouette probability, equal to *c* divided by *b*. All four measures are plotted in order (top to bottom) against time in a single figure. The number of pirouettes initiated and the pirouette initiation probability per frame are smoothed by a rectangular window of 101 frames (a default that can be modified in the code). This essentially provides continuous binning of these measurements throughout the experiment, with a bin size equal to 101 frames.

Plotting Speed Probability Distributions: The ‘Plot Speed Histograms’ option located under the ‘Tracks’ menu can be used to plot speed probability distributions for the open Tracks file (‘Current Tracks File’ option) or for multiple Tracks files selected by the user (‘Select Multiple Tracks File’ option). If you choose to pool speed measurements across multiple experiments (Tracks files), you will be prompted by the software to enter the number of experiments to be analyzed and then to select the Tracks files. The outputs of this function are plots of worm speed probability density and cumulative distribution functions computed for the selected experiment(s).

Estimating the Fraction of Paralyzed Worms: The ‘Analyze Paralysis’ option under the ‘Tracks’ menu can be used to compute the fraction of worms paralyzed in the open Tracks file (‘Current Tracks File’ option) or in multiple Tracks files selected by the user (‘Select Multiple Tracks File’ option). If you choose the multiple Tracks files option, you will need to enter the number of experiments to be analyzed and then select each Tracks file. The output of this function, which includes the fraction of paralyzed worms in the experiment(s), the average speed, the number of tracks analyzed and several additional parameters, are displayed in MATLAB’s command window in table format, and can also saved to an Excel file ‘Analysis Results.xls’. The Excel file is saved in Worm Analyzer’s default directory (see Preferences menu), and analysis results are appended to any existing data in the file. (*Note*: the file must be closed when the paralysis analysis is performed, or MATLAB will not be able to access it.)

***Analysis Preferences**:* Selecting the ‘Preferences’ option located under the ‘Tracks’ menu opens the interface used to define all movie and analysis parameters (Fig. 4).

Analyze & Display Preferences: Determines which track parameters are analyzed and displayed when single tracks are analyzed.

Tracker Preferences: Sets the camera sample (frame) rate and a calibration factor used to convert pixels to mm. The calibration factor is critical for accurate speed calculations and determines track playback speed.

Track Analysis Preferences:

1. Sliding Window for Smoothing Track Data: Size of rectangular window (in # of frames) for smoothing track data. A larger window will produce smoother-looking tracks, but may mask brief locomotion events.
2. Step for Calculating Worm Speed: Size of step (in # of frames) for calculating changes in X and Y coordinates, which are then used to calculate the worms’ instantaneous speed. A value of 1 indicates subsequent frames are used, a value of 2 indicates differences are taken between frames separated by two frames, etc. A larger step size results in smoother estimates of the worms’ speed, but diminishes the “instantaneous” aspect of this estimate.

Pirouette Identification Preferences:

1. Threshold for Pirouette ID: Turning events, also known as pirouettes, are identified using an angular speed threshold – if at any time the angular speed of the worm exceeds this threshold, the worm is deemed to be turning. The default threshold value (110 °/s) was empirically determined as described in Chalasani et al. (2). As this threshold may vary with experimental conditions, you may wish to adjust it to achieve optimal performance of the algorithm for your movies.
2. Min Run Duration: As defined by Pierce-Shimomura et al. (1), pirouettes may be composed of a single turning event (a reversal or an omega bend) or of a bout of turning events punctuated by short runs. We used this definition to implement the automatic pirouette identification algorithm. Thus, the algorithm does not distinguish between reversals or omega bends, and automatically combines turns separated by less than 'Min Run Duration' into a single pirouette. The default value is 6 sec, following Pierce-Shimomura et al. (1).

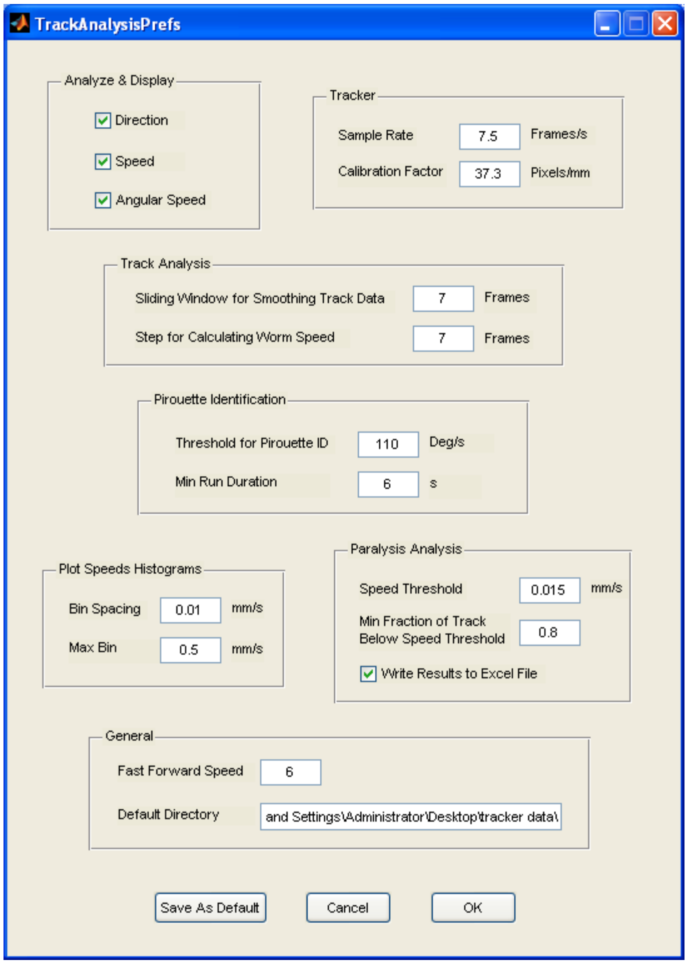

Figure 4: Interface for setting Worm Analyzer preferences

Plot Speeds Histograms Preferences:
Two parameters determine how speed data will be binned to generate speed histograms: the size of individual bins and the maximum bin.

Paralysis Analysis Preferences: Paralysis is assessed by applying a pair of thresholds to the speed measurements associated with each track. A track is classified as paralyzed if the fraction of its speed measurements slower than 'Speed Threshold' is greater than 'Min Fraction of Track Below Speed Threshold'. The default values were chosen to match our hand-scored data. To account for tracks of varying durations, the 'Fraction of Paralyzed Worms' reported by this function is computed by dividing the total duration of paralyzed tracks by the total duration of all tracks in the experiment. If the 'Write Results to Excel File' option is selected, the results of the paralysis analysis are saved into an Excel file, 'Analysis Results.xls', located in Worm Analyzer's default directory (see below). Newly saved data are appended to any existing data in the file. (*Note*: the file must be closed, or MATLAB will not be able to access it.)

General Preferences:

1. Fast Forward Speed: the default fast forward (and rewind) rate is 6x the normal frame rate.
2. Default Directory: this parameter sets the default directory first accessed by Worm Analyzer when a Tracks file is opened or saved. It also determines where the 'Analysis Results.xls' Excel file is saved. *Note:* Make sure to include a backslash at the end of the directory name (as in Fig. 4).

Updating Default Preferences: Analysis Preferences can be saved for future analysis sessions using the 'Save As Default' button. Pressing this button saves all current preferences to the 'Analysis Prefs' worksheet in the Excel file 'Worm Tracker Preferences.xls', which is located in the 'Matlab Functions' folder. (*Note*: the Excel file must be closed, or MATLAB will not be able to access it.) Default preferences can also be directly modified in 'Worm Tracker Preferences.xls' using Excel. We do not recommend this method however, as changes to the spreadsheet may result in tracker failure.

A complete list of the functions comprising the Worm Analyzer package is provided in a separate document, in block diagram form. Each block in the diagram represents a single MATLAB function (M-file); arrows indicate function hierarchy (an arrow points from function A to function B if A calls B).

VII. Video Capture:

To open the Video Capture interface (Fig. 5) type VideoCapture in the MATLAB command window.

Video Capture is designed to capture a sequence of movies of fixed duration at regular intervals. In the example shown in Fig. 5, pressing the 'Start Capture' button will trigger capture of 30 s videos every 10 minutes, for a total of 190 minutes (19 movies in all). To capture a single movie, 'Experiment Duration' should be equal to (or smaller than) the value of 'Capture Every'.

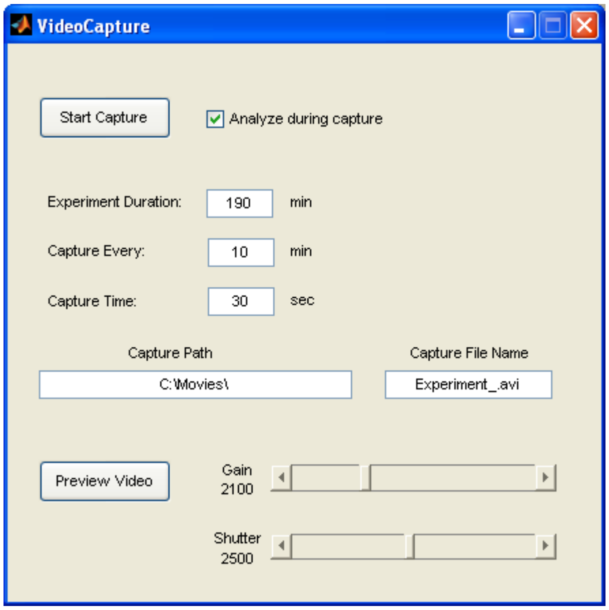

Figure 5: Video Capture interface

As movies are captured, they are saved to the folder specified in the 'Capture Path' box. Serial numbers are appended to the 'Capture File Name' to generate movie names. In Fig. 5, for example, nineteen movies named 'Experiment_1.avi' through 'Experiment_19.avi' will be saved to the folder 'C:\Movies'. *Note:* Make sure to include a backslash at the end of the directory name (as in Fig. 4).

Once the 'Start Capture' button is pressed, its label will switch to 'Stop Capture'. Pressing the 'Stop Capture' button terminates the video capture sequence.

The 'Preview Video' button displays the camera's real-time video input in a preview window. This can be useful for positioning assay plates, adjusting lighting, focus and zoom, and setting the camera gain and shutter speed (using the sliders to the right of the 'Preview Video' button) prior to video capture.

Tracking& analyzing during capture: Selecting the 'Analyze during capture' option automatically activates worm tracking and paralysis analysis for each newly captured video. Similar to Worm Tracker, Tracks files generated in this manner are saved using the name of the movie to which they correspond, with the .avi extension replaced by a .mat extension. Paralysis analysis is identical to the 'Analyze Paralysis' option in Worm Analyzer; results are displayed in MATLAB's command window, and can be saved to the Excel file 'Analysis Results.xls' (as long as that file is closed when the analysis is performed). When capturing a sequence of movies, this feature can be used to track and analyze newly captured movies between capture periods, potentially expediting the data analysis process. However, the time between capture periods must be sufficiently long to allow Worm Tracker to complete tracking; otherwise capture of the next movie will be interrupted.

Video Capture Preferences and Defaults: All Video Capture preferences and defaults are defined in the 'Video Capture Prefs' worksheet in the Excel file 'Worm Tracker Preferences.xls', which is located in the 'Matlab Functions' folder. To modify these defaults, open 'Worm Tracker

Preferences.xls’ using Excel, and overwrite the current values. It is important to ensure that the format of the file and the location of parameters in the spreadsheet are unchanged. Changes to the format or parameter location may disrupt Video Capture initialization.

Analyze during capture preferences: Worm Tracker and Analysis preferences used when the ‘Analyze during capture’ option is selected are the default preferences defined in the ‘Tracker Prefs’ and ‘Analysis Prefs’ worksheets in ‘Worm Tracker Preferences.xls’. To modify these defaults, use either the ‘Save As Default’ option in the Preferences interfaces for Worm Tracker and Worm Analyzer, or directly modify parameter values using Excel.

Configuring Video Capture for additional cameras: Video Capture is designed to work with Sony’s XCD-SX900 digital camera, storing uncompressed, 640×480 grayscale (8-bit) movies in AVI format. It should be possible to configure Video Capture to work with any camera supported by MATLAB’s Image Acquisition Toolbox, however. To check if your camera is supported by the toolbox, please refer to the Mathworks website. If your camera is compatible, you may need to modify the Video Adaptor Name (currently ‘dcam’), the Video Format (currently ‘Y8_1280x960’), and Sample Rate (currently 7.5 frames/s) parameters used by Video Capture. These preferences are defined in ‘Worm Tracker Preferences.xls’ (‘Video Capture Prefs’ worksheet), and should be modified using Excel. For detailed instructions on selecting appropriate values for the Video Adaptor Name and Video Format parameters, please refer to the help files accompanying MATLAB’s Image Acquisition Toolbox. In addition, depending on your camera model, several camera-specific parameters such as maximum and minimum camera gain and shutter speed may need to be modified directly in the code. This will require some familiarity with the MATLAB programming environment.

VIII. Some Final Words:

As mentioned above, we do not have the resources to provide any form of technical support for this software.

Please feel free to modify the software for your own needs. If you develop code that you feel adds functionality to the software and may be useful for other worm researchers out there, please contact us (danielramot-at-gmail.com, mbgoodman-at-stanford.edu). We would like to assist you in distributing your extensions.

As always in science, nothing is ever accomplished quite on one's own. This tracker was no exception. We'd like to thank Ron Milo, currently at Harvard University, for suggesting MATLAB as a useful environment for quickly and efficiently coding a tracker. Thanks also to Cyrus Wilson of the Theriot Lab here at Stanford University for getting us started in the tracker business – his single-worm tracker in C++, the code for which he was kind enough to provide us with, was a great starting point for developing this multi-worm tracker in MATLAB.

IX. References

1. Pierce-Shimomura JT, Morse TM, Lockery SR. The fundamental role of pirouettes in *Caenorhabditis elegans* chemotaxis. *J Neurosci* 19: 9557-9569 (1999).
2. Chalasani SH, Chronis N, Tsunozaki M, Gray JM, Ramot D, Goodman MB, Bargmann CI. Dissecting a circuit for olfactory behaviour in *Caenorhabditis elegans*. *Nature* 450:63-70 (2007)
