# Supplementary figures and images for: The Parallel Worm Tracker: A Platform for Measuring Average Speed and Drug-Induced Paralysis in Nematodes
Source: PLoS One. 2008 May 21;3(5):e2208. doi: 10.1371/journal.pone.0002208 (PMC2373883; doi:10.1371/journal.pone.0002208)

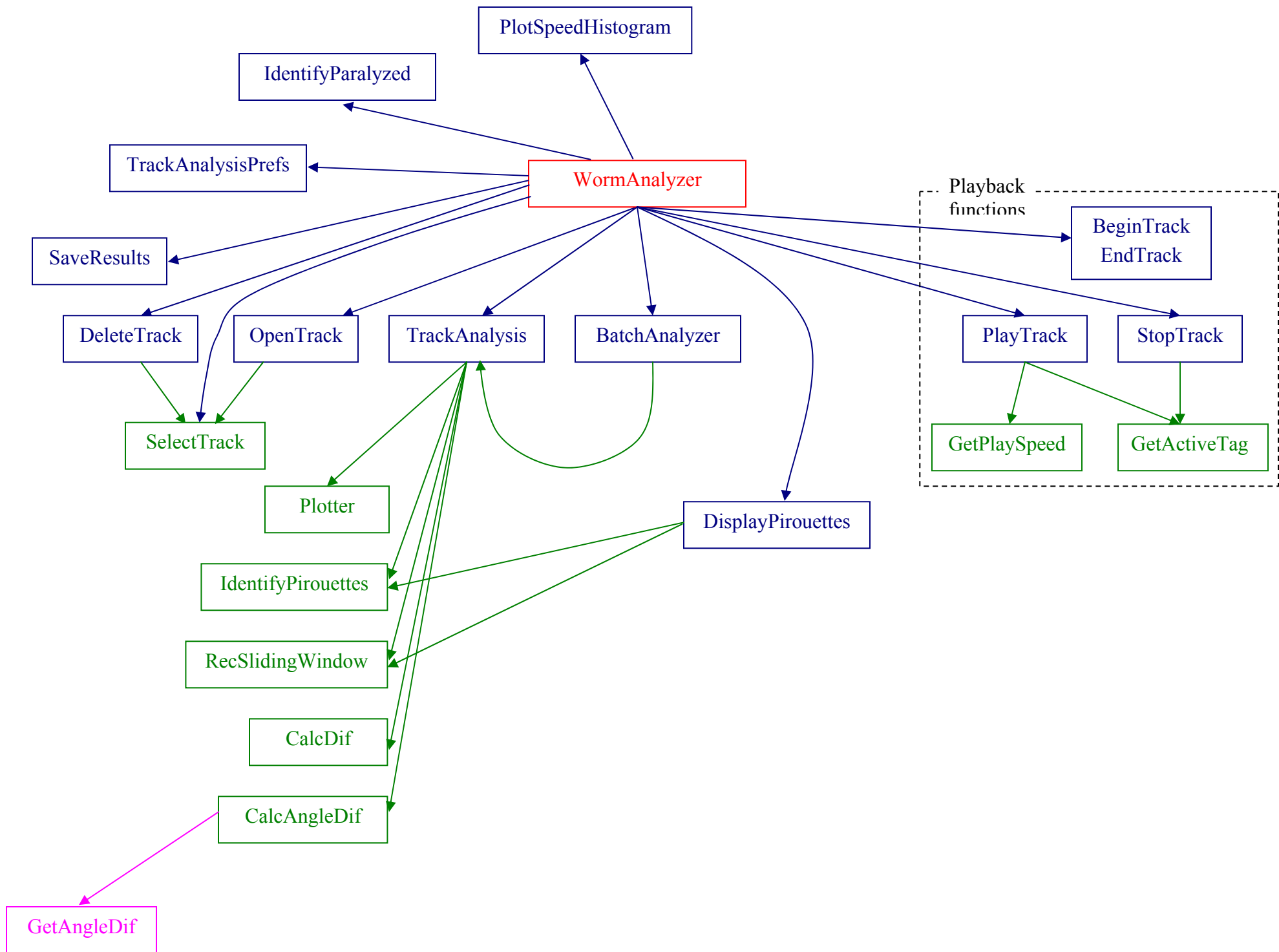

Supplement: Dataset S1 — MATLAB-based code for the Parallel Worm Tracker. This *.zip file contains all of the *.m files needed to run the Parallel Worm Tracker. It also contains the user manual and an Excel file used to define run-time preferences. (0.36 MB ZIP) [file pone.0002208.s001.zip › Track Analysis Functions - Block Diagram.pdf]
